# An Evaluation of Magnetic Resonance Imaging Dixon Sequence Fat–Water Separation Techniques (T2w Dixon FSTs) to Detect Dorso-Lumbar Structural Lesions in Patients with Axial Spondyloarthritis

**DOI:** 10.3390/bioengineering12050502

**Published:** 2025-05-09

**Authors:** David Fadli, Pierre-Francois Lintingre, Laurence Dallet, Julien Raoult, Julien Gay-Depassier, Nicolas Bouguennec, Lionel Pesquer, Benjamin Dallaudière

**Affiliations:** 1 Centre D’imagerie Ostéoarticulaire, Clinique du Sport, Bordeaux Mérignac, 2, rue Georges-Négrevergne, 33700 Mérignac, France; david.fadli.pro@gmail.com (D.F.); julien.raoult@ch-libourne.fr (J.R.); j.gaydepassier@gmail.com (J.G.-D.); 2 Clinique du Sport de Bordeaux-Mérignac, 33700 Mérignac, France; pf.lintingre@imagerie-enosis.fr (P.-F.L.); nbouguennec@gmail.com (N.B.); l.pesquer@imagerie-enosis.fr (L.P.); 3 Centre de Résonance Magnétique, CNRS/University of Bordeaux, 33000 Bordeaux, France; laurence.dallet@rmsb.u-bordeaux.fr; 4 Department of Radiology, University Hospital of Bordeaux, Centre de Résonance Magnétique, CNRS/University of Bordeaux, 33000 Bordeaux, France

**Keywords:** spondyloarthritis, magnetic resonance imaging (MRI), fat, DIXON

## Abstract

Objective: The aim of this study was to assess and compare the diagnostic accuracy of two MRI techniques for identifying structural bone lesions (fatty lesions [BMFs], subchondral erosions [BMEs], sclerosis [BMS], and ankylosis [A]) in the dorso-lumbar joints. This assessment specifically focused on the application of MRI Dixon sequence fat–water separation techniques (T2w Dixon FSTs) when acquiring T1-weighted (T1w) images as the reference standard, among patients diagnosed with axial spondyloarthritis (SpA). Methods: Conducted at a single center, this study involved the recruitment of patients who underwent both spinal and sacroiliac (SI) joint MRI between 2019 and 2022, with follow-up continuing until 2023. In 2023, three independent readers reassessed the initial MRI datasets to evaluate specific radiological features of SpA. They recorded confidence estimates regarding the use of T2w Dixon FSTs when acquiring T1w images. The centralized MRI interpretations were then compared to established rheumatologic diagnoses. Results: A total of 73 patients (42 men and 31 women) were included in the study. The mean sensitivity, specificity, and accuracy of T2w Dixon FSTs (fat-only images) were at least 75%, 100%, and 96%, respectively, based on the 2023 assessment for all considered items. The diagnostic performance of T2w Dixon FSTs was comparable to that of T1w images. Conclusions: The diagnostic performance of T2w Dixon FSTs (fat-only images) matched that of T1w images not only in assessing structural and fatty lesions, but also in the evaluation of subchondral erosions, sclerosis, and ankylosis in the dorso-lumbar joints of patients with a rheumatologic diagnosis of SpA. These findings suggest the potential avoidance of T1-weighted images when employing multi-parameter, multi-sequence imaging, such as the Dixon sequence.

## 1. Introduction

Spondyloarthritis (SpA) presents as a complex inflammatory rheumatologic disease with a diverse clinical spectrum [1], posing diagnostic challenges. The imperative of early diagnosis has escalated for rheumatologists since the introduction of biotherapies in the 2000s. Patients fulfilling the Assessment of SpondyloArthritis International Society (ASAS) criteria can undergo treatment with tumor necrosis factor inhibitors (TNFis) [2] to mitigate the progression of structural damage [3].

Imaging assumes a pivotal role in SpA classification, due to the absence of distinct clinical symptoms and the variable nature of disease activity over time [3]. MRI is increasingly recommended, marking a substantial evolution in the diagnostic approach. MRI’s capability to visualize inflammatory and fat lesions within bone in three dimensions is particularly noteworthy [4]. Hence, the diagnosis of SpA can be achieved earlier through multi-parameter, multi-sequence imaging. Advancement in MRI technology has introduced an array of sequences, including the Dixon sequence—a quantitative measure of tissue water–fat ratio imaging [5]. The Dixon fat–water separation technique is based on multi-echo acquisition and the difference in resonance frequencies between fat and water protons, allowing reconstruction of in-phase, opposed-phase, water-only, and fat-only images. Compared to conventional fat suppression techniques, T2w Dixon offers more homogeneous fat suppression and is less affected by magnetic field inhomogeneities, making it particularly suitable for musculoskeletal imaging.

Inflammatory changes in the bone marrow, such as bone marrow edema, precede structural alterations like fatty lesions (BMFs), bone erosion (BME), sclerosis (BMS), or ankylosis. Quantitative assessment using MRI proves valuable in determining the stage of dorso-lumbar spondyloarthritis. However, there is a limited number of reports on the practical application of the Dixon technique in evaluating bone marrow lesions. Regarding sacroiliac spondyloarthritis, a study in 2021 by Ming-Shan Du et al., involving 45 patients, established that the water–fat ratio of the MRI T2w Dixon sequence can serve as a reference index for evaluating the degree of BME in the active stage of sacroiliac arthritis [6]. Similarly, in 2022, C P Y Chien et al., in a study with 20 patients, observed that the diagnostic performance of fat-only and in-phase images was comparable and superior to that of opposed-phase images [7]. Addressing spinal lesions, a study by Ga Young Ahn et al. in 2021 [8], encompassing 52 patients, explored the role of fat–water separation techniques as an indicator of both active inflammation and chronicity. According to their perspective, this sequence may aid in assessing both SpA disease activity and chronicity, although, regrettably, bone erosions were not evaluated [8].

The objective of this study was to compare the diagnostic efficacy of two distinct sets of magnetic resonance imaging (MRI) in detecting structural bone marrow lesions, including fatty lesions (BMF), subchondral erosions (BMEs), ankylosis (A), or sclerosis (BMS), in the dorso-lumbar joints. This comparison specifically focused on the application of T2w MRI Dixon sequence fat–water separation techniques (Dixon FSTs) when acquiring T1-weighted (T1w) images as the reference standard, in patients with a rheumatologic diagnosis of SpA.

## 2. Materials and Methods

### 2.1. Participants and Study Design

**Figure 1:**
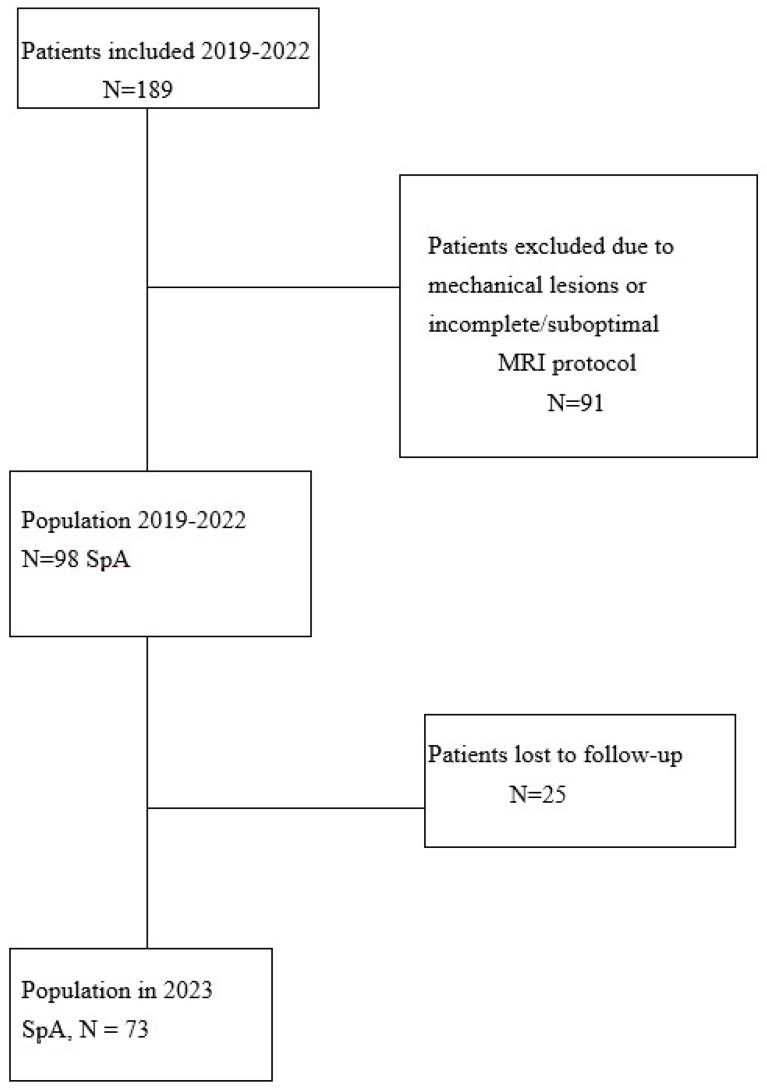
Flowchart of included patients. SpA = spondyloarthritis.

Between 2019 and 2022, consecutive patients with suspected spondyloarthritis (SpA), referred by the rheumatology department to a single center for MRI of the spine and sacroiliac (SI) joints, were prospectively enrolled in the pre-inclusion phase. Eligible participants were those aged 16 years or older and experiencing chronic back pain with an onset before the age of 45 that had persisted for more than three months. Exclusion criteria included contraindications for MRI and a history of spinal or pelvic surgery with metal implants. Following comprehensive follow-up, which included clinical evolution and treatment response assessment in 2023, patients diagnosed with SpA based on clinical and paraclinical criteria aligning with the Assessment of SpondyloArthritis International Society (ASAS) criteria were ultimately included. Demographic, clinical, and biological data were collected at the initial MRI consultation. Ethical approval from the Human Ethics Committee (Institutional Review Board) and informed consent from all participants were obtained for the use of clinical data (Figure 1: flowchart).

### 2.2. Imaging Procedures and Interpretation

MRI examinations were conducted using a 1.5 T magnet (General Electric Artist-MR-750 W). The imaging protocol included coronal oblique T1 and T2-FS sequences of the SI joints and sagittal T1 and T2w Dixon sequences of the thoracolumbar spine (from T7 to S3) (Table 1).

In 2023, three radiologists (J.R, D.F, and B.D, with 2, 5, and 12 years’ experience in musculoskeletal imaging, respectively) re-evaluated the initial MRI datasets at the time of the final diagnosis. They were blinded to the diagnosis and had no access to clinical and biological data. The comparison focused on MRI Dixon sequence fat–water separation techniques (T2w Dixon FSTs) and T1-weighted (T1w) images as the reference standard. The three musculoskeletal radiologists reviewed all MRI datasets in a consensus reading session. In the event of disagreement, findings were discussed until a joint decision was reached. For statistical evaluation of diagnostic performance, individual readings were also assessed independently, and interobserver agreement was calculated using Cohen’s kappa coefficient.

### 2.3. Basis for Interpretation

The water-only images were used as fat-suppressed sequences to evaluate active inflammatory lesions, particularly bone marrow edema, while the fat-only images served as a surrogate for T1-weighted images, allowing improved visualization of structural changes, such as fatty lesions, erosions, sclerosis, and ankylosis. MRI dorso-lumbar joint interpretation with T1w and T2w Dixon FSTs was based on [9].

**Figure 2:**
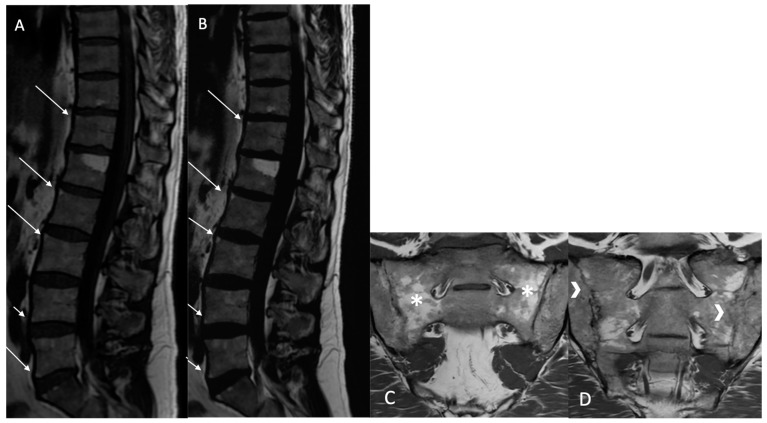
Multiple fatty lesion (BMF) changes and sacroiliac structural changes with partial ankylosis in a 44-year-old patient. A T1w sagittal sequence showing BMF localization at a longitudinal ligamentary structure enthesis (arrows) (**A**). A T2w Dixon (fat) image more clearly showing BMF localization (arrows). No ankylosis, sclerosis, or erosions were found (**B**). A T1w coronal oblique sequence of a sacroiliac joint showing periarticular fatty changes (*). A T1w coronal oblique sequence on a sacroiliac joint showing structural changes with partial ankylosis (arrrowheads). No inflammatory changes were noted (**C**,**D**).

Fatty lesion (BMF): Defined as the presence of high signal intensity, similar to that of adipose tissue, on T1w MR images, or persistent fat areas on the “fat-only” Dixon FST images (Figure 2).

**Figure 3:**
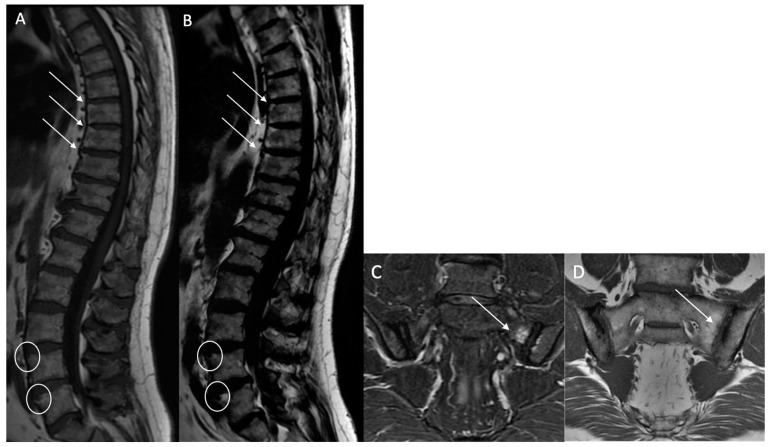
Multiple fatty lesion (BMF) changes and erosions in a 61-year-old patient. A T1w sagittal sequence showing BMF localization mainly at the thoracic longitudinal ligamentary structure enthesis (arrows) (**A**). Focal erosion is noted at the L4 and L5 anterosuperior vertebral corner (circle), and appears as a focal hyposignal. A T2w Dixon (fat) image more clearly showing BMF localization (arrows). Erosion areas are surrounded by fatty replacement areas (**B**). A T2w STIR coronal oblique sequence showing left anterior inflammatory sacroiliitis (**C**). A T1w coronal oblique sequence showing structural changes with an erosive sacroiliac lesion (**D**).

Structural erosions (BMEs): Defined as focal and ill-defined enthesis cortical defects that could be isolated or confluent, with a loss of the normal subchondral cortex appearance and broad irregular margins on both sequences (Figure 3).

**Figure 4:**
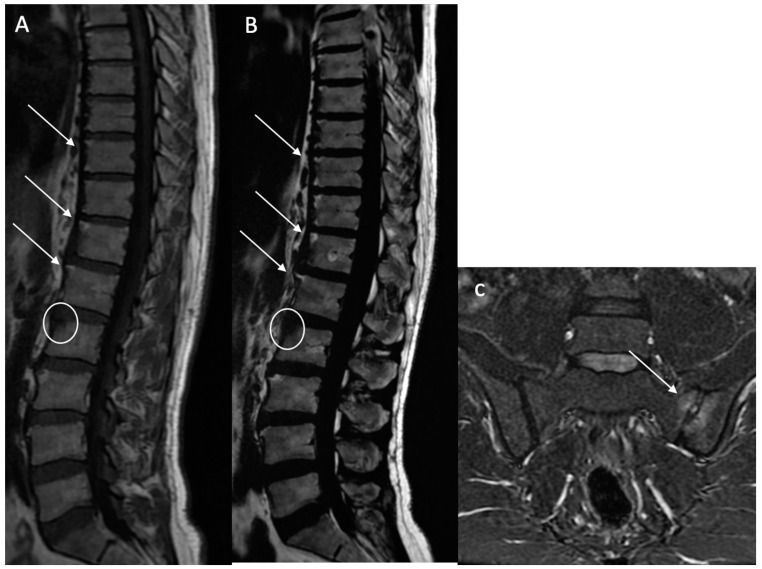
Multiple fatty lesion (BMF) changes and sclerosis in a 55-year-old patient. A T2 Dixon (water) sequence showing thoracolumbar spondylitis and left sacroiliitis. A T1w sagittal sequence showing BMF localization at the longitudinal ligamentary structure enthesis (arrows). Focal sclerosis is noted at the L3 anterosuperior vertebral corner (circle), and appears as a focal hyposignal (**A**). A T2w Dixon (fat) image better showing BMF localization (arrows). Erosion areas are surrounded by fatty replacement areas (**B**). A T2w STIR coronal oblique sequence showing left anterior inflammatory sacroiliitis (arrow) (**C**).

Sclerosis (BMS): Defined as focal, multifocal, or diffuse increased density of the bone matrix, typically showing iso-hypointensity in the skeletal muscle on both sequences (Figure 4).

**Figure 5:**
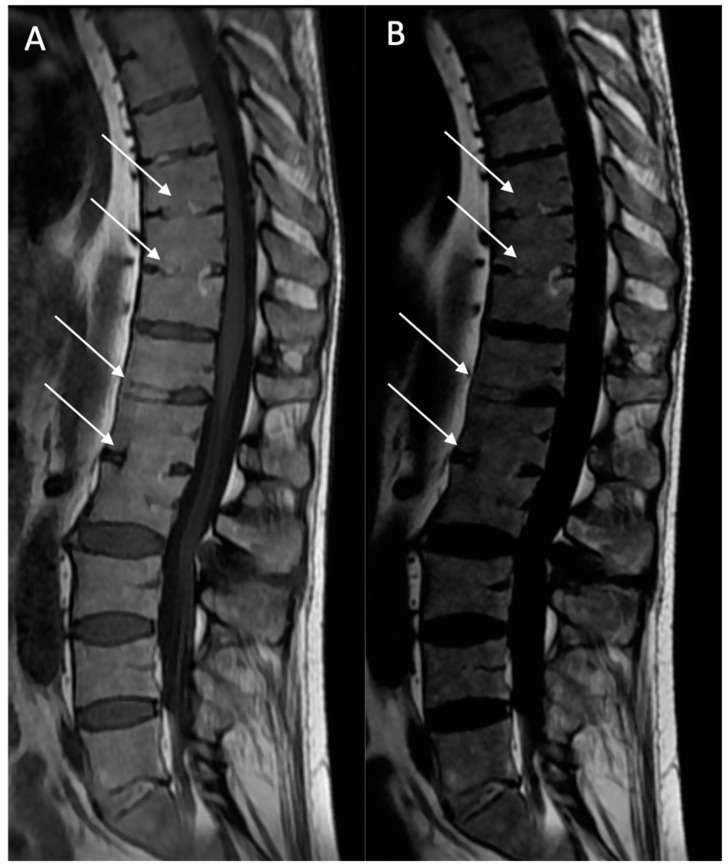
Lumbar vertebral ankylosis with “squared vertebrae” in a 54-year-old patient. A T1w sagittal sequence showing vertebral ankylosis with corporeal vertebral partial bone fusion from T9 to T11 and from T12 to L2 (arrows). The “squared” aspect of the corporeal vertebrae is noted (**A**). A T2w Dixon (fat) image better showing vertebral ankylosis with corporeal vertebral partial bone fusion (arrows) (**B**).

Ankylosis (A): Defined as bony ankylosis with heterotopic bone formation and no discal space on both sequences (Figure 5).

### 2.4. Statistical Analysis

All statistical analyses were performed with R software (R: A Language and Environment for Statistical Computing, R Core Team, R Foundation for Statistical Computing, Vienna, Austria, 2016, https://www.R-project.org).

We evaluated the reliability of T2w DIXON FST sequences for the detection of bone marrow lesion compared to T1w images. The diagnostic performance of MRI data in the detection of lesions were assessed for the whole sample using the final data (after consensus was reached by three readers).

The results from the quantitative variables are presented as the mean ± standard deviation (SD). Those from qualitative variables are expressed in frequency and percentage. For quantitative variables, a *t*-test was performed.

Sensitivity (Se), specificity (Sp), kappa, and accuracy values were estimated for each MRI examination, according to the diagnosis obtained by the rheumatologist. Accuracy, expressed as a percentage, was the overall probability that a patient was correctly classified. This parameter was calculated as follows:

Accuracy = Sensitivity × Prevalence + Specificity × (1 − Prevalence).

Comparisons of MRI performance were made using the area under the curve (AUC). Calculations were performed with R. *p*-values less than 0.05 were considered statistically significant.

The significance threshold selected for all the statistical analysis was 0.05.

## 3. Results

In total, 189 patients with chronic back pain and suspected spondyloarthritis (SpA) were referred by the rheumatology department for MRI examinations between 2019 and 2022. Following comprehensive follow-up and a thorough clinical and paraclinical assessment, 73 patients received a definitive diagnosis of SpA. Notably, 91 patients were eventually diagnosed with another non-inflammatory disease, and 25 were lost to follow-up after the initial MRI examination (Figure 1: flowchart).

The 73 included patients had a mean age of 43.8 years (standard deviation: 12.8; range: 19 to 71), comprising 42 men and 31 women. All patients met the ASAS classification criteria and were diagnosed with axial SpA. The distribution of lesion-affected regions within the dorso-lumbar joints is detailed in Table 2, showing no significant difference in the number of regions between the two sequences.

Table 3 presents the diagnostic performance of both sets of MR images for detecting fatty lesions (BMFs), subchondral erosions (BMEs), sclerosis (BMS), and ankylosis (A). For BME detection, the DIXON FST sequence (n = 18) demonstrated an accuracy rate of 100% (95% confidence interval [81.47–100]). Specifically, 7 patients with BME lesions were correctly identified (sensitivity: 100%), and 11 patients without BME lesions were accurately identified as negative (specificity: 100%).

Regarding BMF detection, the T2w DIXON FST sequence (n = 28) achieved an accuracy rate of 96.43% (95% confidence interval [81.65–99.91]). In this context, 24 patients with BMFs were correctly identified (Sensitivity: 75%), and 3 patients without BMFs were accurately identified as negative (Specificity: 100%). Interestingly, only one patient identified as positive for BMF detection based on the T2w DIXON FST sequence was also positive based on the T1w sequence.

For ankylosis detection, the T2w DIXON FST sequence (n = 6) exhibited an accuracy rate of 100% (95% confidence interval [54.07–100]). In this category, three patients with ankylosis lesions were correctly identified (Sensitivity: 100%), and three patients without ankylosis lesions were accurately identified as negative (Specificity: 100%).

In terms of sclerosis detection, the T2w DIXON FST sequence (n = 5) displayed an accuracy rate of 100% (95% confidence interval [47.82–100]). Specifically, three patients with sclerosis lesions were correctly identified (Sensitivity: 100%), and two patients without sclerosis lesions were accurately identified as negative (Specificity: 100%).

The T2w DIXON FST sequence demonstrated excellent predictability for BME, BMS, and ankylosis (AUC = 1), and very good predictability for BMFs (AUC = 0.875). Observers reported very good agreements between the T2w DIXON FST sequence and the T1w images used as the reference standard for BME, ankylosis, and sclerosis (kappa = 1), and almost perfect agreement for BMFs (kappa = 0.8372).

Importantly, there were no statistically significant changes in sensitivity, specificity, and accuracy values when comparing MRI Dixon sequence fat–water separation techniques (T2w Dixon FSTs) with T1-weighted (T1w) images as the reference standard.

## 4. Discussion

This study found that T2 Dixon FSTs performed comparably to T1-weighted images in the assessment of structural lesions such as fatty changes, bone erosions, sclerosis, and ankylosis in the dorso-lumbar joints of individuals diagnosed with spondyloarthritis (SpA). Specifically, the diagnostic accuracy rate was 100% for detecting erosions, sclerosis, and ankylosis. However, the small number of sclerosis and ankylosis cases in our cohort limits the generalizability of these specific findings. For structural fatty lesions (BMFs), the diagnostic performance was slightly lower due to a lack of specificity. Notably, false positive cases in the T2w DIXON FST (fat-only) sequence for detecting fatty lesions raise questions, given that the standard T1w method used has been demonstrated to be less efficient in detecting fat deposition than the tested “fat-only” T2DIXON FST.

Moreover, considering the overall protocol duration, these data are of particular interest. To our knowledge, there is a limited number of series focusing on MRI Dixon sequence fat–water separation techniques (Dixon FSTs) in axial SpA, especially in the dorso-lumbar joints.

Regarding the sacroiliac joints, literature is significant [10,11,12,13,14,15] but only five series are identified on Dixon technique. In 2017, Özgen et al. explored the value of the T2-weighted multipoint Dixon technique as a single sequence in MRI of the sacroiliac joints for diagnosing active and chronic sacroiliitis. Their findings suggested that the T2-weighted multipoint Dixon sequence is superior to conventional MRI sequences in depicting diagnostic signs of active and chronic sacroiliitis, and may be utilized as a standalone sequence [16].

In 2020, Huang H et al. contemplated the replacement of the standard protocol with qualitative and quantitative T2-weighted Dixon sequence assessment in SpA sacroiliitis. Their study indicated no significant difference in diagnostic performance between the T2W Dixon sequence and the standard protocol. The single T2W Dixon sequence showed potential to replace the standard protocol in patients with suspected axial SpA, offering a potential reduction in acquisition time [17].

In 2021, Ming-Shan Du et al. explored bone marrow edema (BME) in the sacroiliac joints of patients with ankylosing spondylitis using magnetic resonance imaging (MRI) Dixon sequences. They established that the water–fat ratio obtained from the MRI Dixon sequence serves as a valuable reference index for evaluating the extent of BME during the active stage of sacroiliac arthritis. Notably, the water–fat ratio beneath the bilateral sacroiliac joints on Dixon sequence images in the case group significantly exceeded that in the healthy control group (*p* < 0.05). Furthermore, the Dixon sequence water–fat ratio on the iliac and sacral surfaces of the bilateral sacroiliac joints in the study group exhibited a positive correlation with spinal arthritis research [6].

Similarly, in 2021, Crema et al. focused on detecting subchondral erosions in the sacroiliac joints using T1-weighted fat-suppressed MRI in 31 patients. They compared the diagnostic performance of two different MRI sets for detecting subchondral erosions in the sacroiliac joints, considering the application of fat–water separation techniques during T1w acquisition. The assessment of T1w images with fat suppression significantly enhanced sensitivity, specificity, positive predictive value, and overall accuracy in detecting erosions when compared to assessments without fat suppression [18].

In a more detailed exploration in 2022, C P Y Chien et al. conducted a study involving 20 patients, delving into the impact of subchondral edema in T2-weighted Dixon MRI sequence evaluation of sacroiliac joint erosion in axial spondyloarthropathy. Their findings indicated that the diagnostic performance of fat-only and in-phase images was similar, and superior to that of opposed-phase images. The interobserver reliability of fat-only and in-phase images showed substantial and moderate agreement, respectively, when compared to opposed-phase images. In the subgroup analysis, the specificity and AUC for the oedema-positive group were lower than the oedema-negative group in all image sets. Interobserver reliability was substantial for fat-only and in-phase images in both groups, but slight and moderate for the opposed-phase oedema-positive and negative groups, respectively. The presence of subchondral edema in active sacroiliitis decreased the diagnostic accuracy of sacroiliac joint erosion detection on T2W Dixon MRI [7].

In a recent study in 2023, Martín-Noguerol et al. confirmed these results, emphasizing the crucial role of advanced MRI techniques in assessing and quantifying sacroiliitis, offering promising and robust outcomes [5].

Regarding dorso-lumbar joints, only one series was found on PubMed. In 2021, Ga Young Ahn et al. [8] investigated the role of fat–water separation techniques as indicators of both active inflammation and chronicity in 52 patients. They suggested that this sequence may aid in assessing both AS disease activity and chronicity, although, unfortunately, bone erosions were not assessed [8].

It is important to note that our study has certain limitations. The clinical (rheumatologic) reference standard may be imperfect, allowing for the possibility of inaccuracies in the final diagnosis in some cases. However, efforts were made to mitigate this bias by conducting a long-term follow-up with a substantial number of participants at baseline and follow-up. Another limitation is that MRI readings were centralized in a department specializing in musculoskeletal imaging, which may not fully represent real-world experiences.

In conclusion, the diagnostic performance of T2w Dixon FSTs was found to be comparable to that of T1w images in assessing structural lesions in the dorso-lumbar joints with a rheumatologic diagnosis of SpA. These findings suggest that T2-weighted Dixon fat–water separation sequences may serve as a valid alternative to T1-weighted imaging for the detection of structural lesions in the dorso-lumbar spine, when used within a multi-sequence MRI protocol. However, further validation in larger and more diverse cohorts is needed before recommending routine omission of T1-weighted sequences.

## Figures and Tables

**Table 1:** MRI protocol.

	Repetition Time (ms)	Echo Time (ms)	Number of Signals Acquired	Slices Thickness (mm)	Field of View (cm)	Acquisition Time (min·s)
Coronal oblique STIR of SI joints	5206	50	1.5	3	26	2.26
Coronal oblique T1-weighted of SI joints	595	minfull	1	3	26	1.09
Sagittal T2 DIXON of thoracolumbar spine	3058	90	2	3.5	32	2.58
Sagittal T1-weighted of thoracolumbar spine	450	minfull	1	3.5	32	1.34

Note—SI = sacroiliac.

**Table 2:** The total number of regions exhibiting lesions at each of the locations of the dorso-lumbar joints.

Lesion	T1w Sequence	T2w DIXON FST Sequence	*p*-Value
Fatty lesion (BMF)	176	256	*p* = 0.1055
Subchondral erosion (BME)	17	13	*p* = 0.7086
Sclerosis (BMS)	2	2	*p* = 0.7113
Ankylosis (A)	3	3	*p* = 0.4857

**Table 3:** MRI performance of T2w DIXON FSTs in the detection of lesions, using T1w as the reference standard for spondyloarthritis.

	Accuracy (CI)	Sensitivity	Specificity	AUC
Fatty lesion (BMF)	96.43% [81.65–99.91%]	100%	75%	0.875
Subchondral erosion (BME)	100% [81.47–100%]	100%	100%	1.00
Sclerosis (BMS)	100% [47.82–100%]	100%	100%	1.00
Ankylosis (A)	100% [54.07–100%]	100%	100%	1.00

Percentages are rounded. Numbers in brackets are limits of 95% confidence intervals. AUC = area under curve.

## Data Availability

The data presented in this study are available on request from the corresponding author.

## Abbreviations

The following abbreviations are used in this manuscript:

MRI	Magnetic resonance imaging
Dixon FST	Dixon sequence fat–water separation technique
BMF	Bone fatty lesion
BME	Bone subchondral erosion
BMS	Bone structural sclerosis
A	Ankylosis
SpA	Spondyloarthritis
T1w	T1-weighted
Se	Sensitivity
Sp	Specificity
